# Blocking the Trigger: Inhibition of the Initiation of Bacterial Chromosome Replication as an Antimicrobial Strategy

**DOI:** 10.3390/antibiotics8030111

**Published:** 2019-08-06

**Authors:** Julia E. Grimwade, Alan C. Leonard

**Affiliations:** Program in Biological Sciences, Department of Biomedical and Chemical Engineering and Sciences, Florida Institute of Technology, 150 West. University Blvd., Melbourne, FL 32901, USA

**Keywords:** antibiotic targets, orisome, *oriC*, DnaA, DnaB, DnaC, initiation of bacterial DNA replication

## Abstract

All bacterial cells must duplicate their genomes prior to dividing into two identical daughter cells. Chromosome replication is triggered when a nucleoprotein complex, termed the orisome, assembles, unwinds the duplex DNA, and recruits the proteins required to establish new replication forks. Obviously, the initiation of chromosome replication is essential to bacterial reproduction, but this process is not inhibited by any of the currently-used antimicrobial agents. Given the urgent need for new antibiotics to combat drug-resistant bacteria, it is logical to evaluate whether or not unexploited bacterial processes, such as orisome assembly, should be more closely examined for sources of novel drug targets. This review will summarize current knowledge about the proteins required for bacterial chromosome initiation, as well as how orisomes assemble and are regulated. Based upon this information, we discuss current efforts and potential strategies and challenges for inhibiting this initiation pharmacologically.

## 1. Introduction

Since the 1940s, antibiotics have become an integral part of global health care, and combined with advances in food handling, sewage treatment and other improvements in general hygiene, antibiotics have led to a dramatic decrease in mortality and morbidity caused by bacterial infections [1]. The ability to control bacterial infections has also stimulated advances in surgical techniques and chronic disease management [2]. However, the use of antibiotics is accompanied by a rise in the number of drug-resistant bacteria in the environment. The emergence of antibiotic resistance is attributed largely to an overuse of antibiotics in human medicine and in agriculture, which puts enormous selective pressure upon bacterial populations, such that only those bacteria that have resistance mechanisms can survive [2]. Resistance comes from mutations in the bacterial molecular target of the drug, and by acquisition, via the horizontal gene transfer (HGT), of genes that can inactivate or remove the drugs from the bacterial cell [1]. HGT, in particular, has allowed the emergence of the so-called “Superbugs”: Bacteria that are resistance to multiple antibiotics. The origin of all transferred resistance elements is still speculative; however, antibiotic resistance determinants have been discovered in ancient DNA samples [3,4]**,** and there is evidence that at least some of the resistance genes originated in antibiotic producers as part of their survival strategy [5,6]. Resistance determinants may have also evolved in non-producers to allow them to survive in environments shared with antimicrobial producers [7].

The prevalence of antibiotic-resistant bacteria has resulted in a global health crisis, and there is an urgent need for new antibiotics to treat resistant infections. Logically, when developing these new drugs, effort should be made to delay any development of resistance to them. However, many recent drug discovery efforts have focused upon modifying existing scaffolds [8], and any new agents that are developed in this way risk being inactivated by the same mechanisms that affected the parental compounds. Furthermore, although microbial extracts have been a valuable source of antibiotics in the past, all antibiotics from natural sources risk inactivation by the resistance mechanisms originally used by the antibiotic producers, which can be transferred to pathogens via HGT. Therefore, although it is tempting to continue with past antibiotic discovery strategies, if we wish to avoid a rapid development of resistance, it may be advantageous to avoid pre-existing resistance elements by broadening the search to new areas. For example, since current antibiotics target only a handful of the approximately 300–400 essential bacterial genes [9,10,11], one solution to developing new, effective antibiotics is to target novel pathways. It might also be advantageous to examine pathways that are normally avoided by microbial antibiotic producers.

One unexploited and ecologically-avoided pathway is the one that triggers an initiation of bacterial chromosome replication. This initiation is mediated by the assembly of the orisome complex that unwinds DNA in the unique chromosomal replication origin (*oriC*) and prepares for the establishment of new replication forks. In this review, we summarize current knowledge about orisome assembly and the initiator proteins used in this process. We also discuss progress on identifying initiation inhibitors, and discuss new strategies for targeting replication initiation.

## 2. Proteins Involved in Initiation of Bacterial Chromosome Replication

### 2.1. Overview

Origin DNA strand separation, followed by the loading of two opposing molecules of replicative DNA helicase, are prerequisites for starting new replication forks in all bacteria [12]. Based on studies using *Escherichia coli*, these activities require three proteins: DnaA, the primary initiator protein; DnaB (using *E. coli* nomenclature), the replicative helicase; and DnaC, the helicase loader. These are described in more detail below.

#### 2.1.1. DnaA

**Figure 1:**
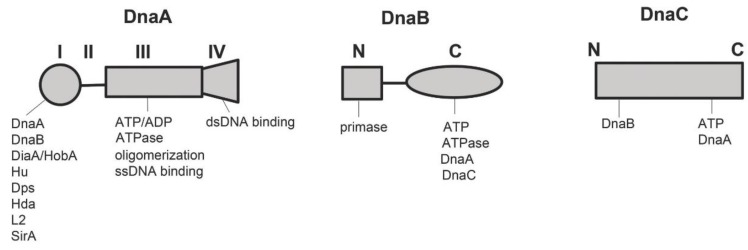
Functional regions of DnaA, DnaB and DnaC. The domain names are shown above the cartoons. Interacting proteins, cofactors and other functions of each domain are shown below the cartoon. See the text for details.

DnaA is a highly conserved protein that has been identified genetically and biochemically as the primary initiator protein in eubacteria [13,14,15]. DnaA is the primary chromosomal initiator protein in almost all bacteria studied, although some bacteria, such as *Vibrio cholerae*, have two chromosomes, and contain distinct and unrelated initiators for the second chromosome [16]. DnaA is responsible for binding to specific sequences in *oriC* and unwinding an A-T rich region termed the DNA Unwinding Element (DUE) [17]. In many bacterial types, DnaA also recruits DnaB and DnaC, and helps position the helicase onto the newly-formed single strands of the unwound DUE [12]. DnaA accomplishes its tasks as an initiator using four domains, each with specific functions, described below and in Figure 1.

The N-terminal domain, domain I, in most bacteria, interacts with regulators of initiation, including the positive regulators DiaA (*E. coli*)/HobA (*Helicobactor pylori*) [18,19] and HU (*E. coli*) [20], as well as the negative regulators Dps (*E. coli*) [21], Hda (*E. coli and Caulobacter crescentus)* [22], ribosomal protein L2 [23], and SirA (*Bacillus subtilis*) [24]. In addition, domain I interacts with helicase DnaB, to recruit it to the origin in some bacterial types [25]. However, in other bacteria (e.g., *B. subtilis*) an additional initiation protein (DnaD) interacts with DnaA, and recruits proteins (*Bs*DnaB, DnaI) that interact with the helicase (termed DnaC in *B. subtilis*) [26,27]. Finally, domain I is also one of the two self-oligomerization domains of DnaA (the other being Domain III, discussed below) in many, but not all bacteria [28,29]. In *E. coli*, this domain I-mediated oligomerization is required for bound DnaA to recruit and position a new DnaA monomer for binding to *oriC* [30] (see Section 3 below).

Domain II is the least conserved of the four domains, and greatly varies in length among eubacterial DnaAs. For example, the DnaA in the thermophilic bacteria *Aquifex aeolicus* has essentially no Domain II, while Domain II in *Streptomyces coelicolor* contains approximately 250 amino acids [29]. This domain has generally been considered to be a flexible linker between domains I and III, and may play a role in determining the distance over which a DnaA monomer that is recruited by domain I of a bound DnaA can be extended [31] (see Section 3 below). If this is the case, then perturbed cooperative binding could explain why the deletion of some of the amino acids in this region decreases the initiation frequency [32].

Domain III, with domain IV (see below) are the most conserved domains in eubacterial DnaA [33,34]. Domain III establishes DnaA as a member of the AAA+ family of ATPases [35], containing motifs involved in the binding of ATP and ADP, and in ATP hydrolysis [15,36]. The ATP-bound form of DnaA is required for the formation of functional orisomes at most (perhaps all) bacterial chromosomal replication origins [25,35], although active *E. coli* orisomes may contain some DnaA-ADP [37,38,39], and DnaA-ADP also shows activity in some plasmid replication systems [40]. In *E. coli*, one major reason that DnaA-ATP is required is to allow the initiator to fully occupy *oriC* [39], because a subset of the lower affinity recognition boxes in the origin preferentially bind this form [41,42]. However, if the sites are modified to allow the access of DnaA-ADP, then DnaA-ADP appears to be capable of performing the mechanical activities required for origin activation [39]. Interestingly, a number of temperature-sensitive DnaA alleles carry a mutation that prevents the binding of both ATP and ADP [43,44]; however, despite the absence of nucleotide binding, the alleles are active, and sometimes over-active (in the case of *dnaA(cos*)) at permissive temperature.

Domain III is also, like domain I, responsible for self-oligomerization [45]. Interaction between the domain III regions of two DnaA molecules requires at least one of the molecules to be bound to ATP [46]. Specifically, the arginine finger motif in one domain III associates with the bound ATP of the other DnaA monomer. Multiple DnaA-ATP monomers can join together in this way to form a helical filament, with additional amino acids in the interface region making contacts that stabilize the oligomer [44,46]. The DnaA-ATP filament contains a central channel which is capable of binding to single-stranded DNA [44,47], possibly at the newly identified DnaA-trio elements found in many bacterial DUE regions [48].

Domain IV is the C-terminal domain, and carries the helix-turn-helix motif responsible for double-stranded DNA binding. In most bacteria studied thus far, DnaA binds to a 9-mer sequence; the consensus recognition sequence for *E. coli* is 5′-TTATCCACA, commonly termed the R-box. [49]. Amino acid residues in domain IV make base-specific hydrogen bonds and Van der Waals contacts at several nucleotides in this sequence, as well as non-specific contacts with the phosphate backbone, in both the major and minor grooves [34,50]. DnaA can also bind to sequences deviating from consensus, although changes of more than 2 bp cause loss of base-specific contacts and a decreased affinity [31,51,52]. As mentioned above, some, but not all, of the non-consensus sites preferentially bind DnaA-ATP [39,41], and binding to these sites also requires interactions between the domain III regions of two bound DnaA-ATP molecules [42]. 

Cryptic lower affinity DnaA recognition sites are not exclusive to *E. coli oriC*, and have been identified in several different bacterial *oriC*s, including *C. crescentus* and *H. pylori* [53,54,55]. Based on this, it has been suggested that cryptic DnaA recognition sites may be a common feature of bacterial origins [53], but because binding studies are often required to identify them, it is not known how prevalent they are in the bacterial world.

#### 2.1.2. DnaB and DnaC

DnaB, the replicative helicase in *E. coli*, is also an ATPase that, when bound to ATP, assembles into a hexameric toroidal ring [25]. Most bacteria contain helicases similar to DnaB, but the nomenclature may differ (e.g., in *B. subtilis* and some other Gram positive bacteria, the replicative helicase is termed DnaC) [56]. DnaB monomers contain two major domains (N-terminal and C-terminal) connected by a short flexible linker [25] (Figure 1). The C-terminal domain of each DnaB monomer in the ring binds adenine nucleotide, harbors the ATPase activity that allows the translocation of helicase along the DNA, and interacts with the N-terminal region of the helicase loader, DnaC [57,58] (Figure 1. Six molecules of DnaC-ATP form a complex with DnaB in the hexameric ring, and alter the DnaB structure to crack open the ring, allowing the helicase to encircle single-stranded DNA at the replication origin [27,59,60,61]. Two opposing DnaA hexamers are loaded onto the unwound origin. In *E. coli*, DnaB is recruited to the origin by an interaction between the N-terminal region of *oriC*-bound DnaA [62] and the C-terminal region of DnaB [63]. The AAA+ region in domain III of DnaA may also play a role in guiding DnaB to the unwound region [64]. Once the binding of helicase to DNA is accomplished, the ATP that is bound to DnaC is hydrolyzed, and the helicase loader dissociates from the complex [65]. This activates each DnaB hexamer to move and expand the unwound DNA bubble [65]. Once loaded and free of DnaC, DnaB helps recruit DNA primase (DnaG) [66,67] via interactions with DnaB’s N-terminal domain [68]. After DNA replication begins, DnaB translocates with each replication fork [25,61,69].

Analysis of the *A. aeolicus* DnaC structure reveals that it, like DnaA, is a member of the AAA+ family of ATPases, and DnaC-ATP shares DnaA’s ability to form a right-handed helical filament via the interactions between the AAA+ domains [70]. The complex of DnaA-DnaC-DnaB is proposed to position one of the DnaB hexamers within the unwound region of *oriC* [70], and in *E. coli*, DnaA bound to the right half of *oriC* has been implicated in positioning the other helicase ring [47]. Some Gram positive bacteria, such as *B. subtilis* and *Staphylococcus aureus*, have alternative helicase loaders. For example, in *B. subtilis*, DnaD, DnaB (not equivalent to *E. coli* DnaB) and DnaI all load helicase [71], and it was suggested that the mechanism of helicase ring opening may also differ in these bacteria [27]. Additionally, some bacteria, such as *H. pylori* and *Pseudomonas sp*. seem to lack a distinct helicase loader [72] similar to DnaC or DnaI. In these bacteria, the loader function may be incorporated into the replicative helicase structure [73], or another protein, such as DciA in *Pseudomonas aerugenosa*, may act as the loader [74].

## 3. Orisome Assembly

**Figure 2:**
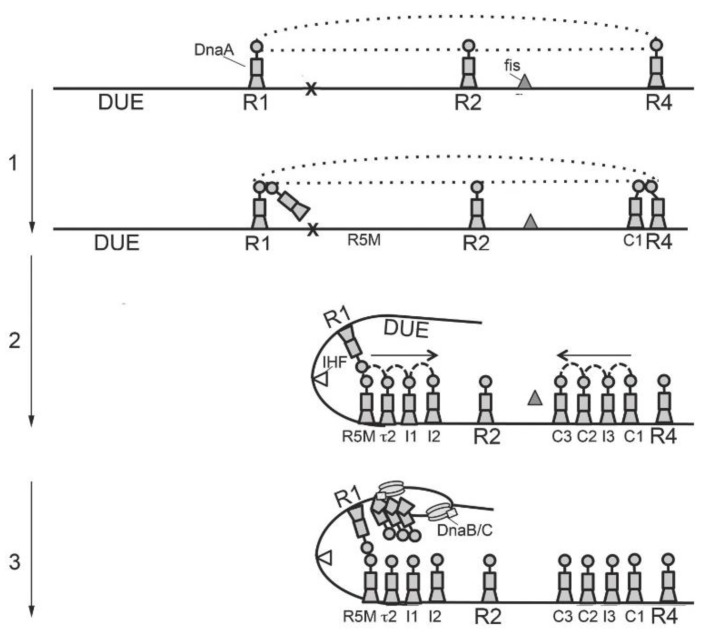
Model of orisome assembly in *E. coli*. Stage 1: DnaA is bound to the high affinity site R1, R2 and R4. The *Escherichia coli* gene encoding the FIS Protein (Fis) is also bound. Interactions between the bound DnaA molecules (shown by the dotted line) constrain the origin and this prevents the integration host factor (IHF) from binding to its recognition site (shown by X). Low affinity sites are unoccupied. DnaA bound to R4 and R1 recruits DnaA for binding to their proximal site, but the lack of IHF-induced bending prevents donation from R1. Stage 2: Progressive cooperative binding of DnaA in the right region of the chromosomal replication origin (*oriC*) (indicated by the dashed arches) results in the occupation of the low affinity I3, C2 and C3 sites, and a displacement of Fis. This allows IHF to bind, and the induced DNA bend allows an interaction between R1 and R5M, followed by a progressive DnaA binding to the *oriC*’s left region. Arrows indicate the direction of progressive binding. Stage 3: Full occupation of double-stranded DNA recognition sites in *oriC* by DnaA results in strand separation in the DNA Unwinding Element (DUE). A DnaA filament binds the single-stranded DNA and recruits DnaB (helicase) and DnaC (helicase loader). See the text for details.

DnaA, in association with *oriC*, assembles into the orisome complex, which unwinds the origin DNA and recruits the DnaB/C complex (Figure 2). The instructions for orisome assembly are carried in bacterial *oriC*s in the form of precisely positioned recognition sites that direct DnaA binding [31,75]. Orisome assembly is most thoroughly characterized in *E. coli* [76,77], where it begins immediately after each initiation event, when three molecules of the initiator DnaA interact with the three high affinity R boxes (R1, R2, and R4) found in *E. coli*’s *oriC* [78] (Figure 2). This binding persists throughout the entire bacterial cell cycle [79,80], forming a scaffold that constrains the origin to prevent any spontaneous unwinding of the inherently unstable DNA Unwinding Element (DUE) region [81]. This scaffold also prevents the simultaneous binding of two DNA bending proteins, the *E. coli* gene encoding the FIS Protein (Fis) and the integration host factor (IHF) to *oriC* [82]. Although it is not known how this constraint is lifted, it is likely that contacts among the three tightly bound DnaA molecules are broken when the bound DnaA recruits additional DnaA molecules using Domain I interactions [30], or when Fis is displaced from *oriC* (see below). 

The recruited DnaA is positioned for binding to the nearest lower affinity site [30] (Figure 2). This recruitment is essential, since lower affinity sites are not capable of binding DnaA independently [30,31,52]. The positioning of DnaA is limited by distance, perhaps determined by the length of DnaA’s domain II [31]. In *E. coli*, the distance between the high affinity site (R1), and the nearest weak site (R5M) in the left region of *oriC* exceeds the allowable distance, and so DNA bending, assisted by IHF, is required to bring the two sites into proximity [31,51]. However, IHF is unable to facilitate this cooperative binding until Fis is displaced (see below) [81]. Either DnaA-ATP or DnaA-ADP is capable of completing orisome assembly up to this stage [39,82]. After this point, the cooperative binding of DnaA-ATP is required to fill the remaining low affinity sites [39].

DnaA-ATP levels increase as cells approach the time of initiation [83], allowing the binding of this form of DnaA to progress toward the center of *oriC*, guided by arrays of closely spaced (2 bp) low affinity recognition sites in each half of *oriC* [31]. Because Fis inhibits the IHF-induced bending necessary for interactions between R1 and R5M sites, the right array of sites fills first and displaces Fis. IHF can now bind, and the resulting bend allows DnaA bound at R1 to recruit and position a new DnaA molecule at R5M. Once R5M is filled, cooperative binding proceeds until the left array is occupied [77,81]. The sites in each array are oriented in the same direction, but the arrays in each half of *oriC* are opposed, so that the arginine finger in domain III of each newly bound molecule of DnaA-ATP faces the center of *oriC* [31,82]. The DnaA-ATP requirement at this stage is caused by the preference of the low affinity sites for DnaA-ATP [39]. 

When all of the sites in the origin are occupied, the AT-rich DNA in the DUE is unwound [17,51]. The mechanism of unwinding is not yet clearly understood, although several models have been proposed [39,46,84,85,86,87].

Once the DNA is unwound, DnaA-ATP associates with the single-stranded region [87,88,89]. DnaA-ATP is required for this step because binding to single-stranded DNA requires the assembly of a helical filament. The central channel of the filament interacts with single-stranded DNA [44]. There are conflicting reports about whether or not specific DNA sequences are needed for this binding [44,87,89], but in *B. subtilis*, and most likely other bacteria, tri-nucleotide “DnaA-trio” elements, with the preferred sequence 5′-g/aAT, bind the DnaA filament [48]. Since DnaA-ADP can not form this filament [44], it presumably is not able to participate in single-stranded DNA binding. Once bound to the DUE, DnaA-ATP then recruits the replicative helicase and the helicase loader (DnaB and DnaC, respectively, in *E. coli*) [62,70].

After initiation, either translocation of helicase or movement of the replication forks through *oriC* is likely to remove the bound DnaA. DnaA rebinds immediately to the three high affinity sites, but any further progression of orisome assembly is blocked by SeqA, which binds to hemi-methylated 5′-GATC sequences in *oriC* and prevents DnaA from interacting with lower affinity sites [80,90]. The Hda protein, associated with the *beta*-clamp of the polymerase in replication forks, stimulates the hydrolysis of ATP bound to DnaA [91]. This causes a rapid lowering of DnaA-ATP levels, preventing the re-occupation of DnaA-ATP sites, so orisome assembly is prevented until new DnaA-ATP is generated [92].

Much less is known about orisome assembly in other bacterial types, in part, because the process has been characterized in only a few bacteria, but also because orisomes are built using a diverse set of instructions; there is little similarity among different bacterial *oriC*s. The consensus R box sequence identified in *E. coli* [49] is utilized by many, but not all, bacteria [52,93,94], and most bacterial origins contain clusters of R-box-like sequences. However, there is surprisingly high diversity in the number, relative position and orientation of recognizable R boxes among different *oriC*s [93,95,96]; for example, the site arrays in the *E. coli oriC* that direct orisome assembly, and the DnaA-ATP sites in these arrays, have not yet been found in other bacterial origins. This could be because cryptic sites in general are not well characterized, but it may be that these are not common features of bacterial origins. Unfortunately, it is also not clear how origin diversity affects orisome assembly pathways, although there is ample evidence that the configuration of a bacterial replication origin is optimized for directing complex assembly using its own DnaA. For example, even though both *E. coli* and *B. subtilis* DnaA proteins bind the consensus R box sequence in vitro and create similar multimeric structures when visualized by electron microscopy, neither *E. coli* nor *B. subtilis* DnaA can unwind the other’s origin. *B. subtilis* DnaA is toxic to *E. coli* cells, probably because in these cells, both EcDnaA and BsDnaA bind to the origin sequences, but the mixed complexes are unable to form the correct oligomeric configuration for function [97]. Similarly, while both *E. coli* and *Mycobacterium tuberculosis* DnaAs bind well to *S. coelicolor oriC*, neither can bend the origin into the structure formed by the native DnaA protein [75,98]. Although it seems logical that orisomes built using a highly conserved initiator protein would contain conserved subcomplexes used for the mechanical aspects of origin activation, there is currently not enough information on different orisomes to be sure that this is true, although there has been some progress this area [93]. Inhibition of orisome assembly after initiation also does not appear to be conserved, since many bacteria lack homologs to Hda and SeqA [77].

## 4. Inhibitors of Replication Initiation: Where We are and Where We might Go

### 4.1. Overview 

While the current lack of knowledge on diverse orisome assembly pathways could discourage the exploitation of this process for drug development, it should be noted that the major initiation proteins have been well-characterized in multiple bacterial types [99], and there are in vitro biochemical assays available that could allow an identification of compounds that specifically target an individual protein or even a specific region of a protein. 

Recent efforts along these lines are discussed below in Section 4.2. However, one must recognize that a limitation of any in vitro assay is that compounds could be identified without any consideration of bacterial permeability, and so any leads must be tested further to ensure that the drug can be delivered to its intracellular target. For this reason, historically the most successful antibiotic discovery platforms have utilized cell-based assays [11]. A few cell-based assays to identify the inhibitors of initiation have been reported [100,101,102,103,104] and are described in Section 4.3. Further, it seems likely that a strategy for inhibiting initiation could be derived from the current understanding of orisome assembly and regulation in different bacteria, and this is discussed in Section 4.4.

### 4.2. In Vitro Screens Targeting DnaA and DnaB

DnaA binds to ATP immediately after it is made in cells, and DnaA-ATP is considered to be the active form of the initiator based on seminal studies of in vitro *E. coli* DNA replication by the Kornberg lab [105]. Thus, in early efforts to look for “druggable” aspects of DnaA structure, the ATP binding cleft emerged as one feature that could be inhibited by small molecule compounds. A small-scale search for synthetic compounds that inhibited ATP binding to DnaA found that 3-acetoxy-2,2′-bi-1H-indol and the derivatives of this compound inhibited both ATP binding and in vitro replication of an *E. coli oriC* plasmid [106]. While there is no evidence that the active indols were used as lead compounds to develop a novel antibiotic, the study does suggest that it is possible to pharmacologically inhibit the binding of ATP to DnaA. However, although it is widely accepted that all other bacteria share the DnaA-ATP requirement for initiation, this property remains uncharacterized in many bacterial types, and a recent study demonstrated that DnaA-ADP or DnaA free of nucleotide could activate a version of *E. coli oriC* engineered so as allow access of these forms to all origin DnaA recognition sites [39]. Further, some of the temperature-sensitive DnaA alleles, including *dnaA(cos)* and *dnaA*46, do not bind nucleotide [43], but still retain activity at permissive temperature, although the mechanism allowing activity is not well understood. Even though it is clearly possible to inhibit ATP binding to DnaA, further study will be needed to determine if this is an effective or sensible mechanism of action for a new antimicrobial agent.

Bacterial replicative helicases are considered promising drug targets based on their essential nature in bacterial replication, the availability of structural data for several different bacterial helicases, and the fact that they differ sufficiently from their eukaryotic counterparts to allow them to be specifically targeted, minimizing the toxicity of any developed agents [99,107,108]. Several screens identified natural products in the flavinol family (e.g., myrecetin and galangin) that inhibit the DNA unwinding activity of *E. coli* and *Klebsiella pneumoniae* DnaB proteins [109,110]. Although the precise mechanism of inhibition was not entirely clear, there was evidence that the binding of ATP and also the ATPase activity could be affected [111], a result that may encourage further efforts at finding inhibitors to these specific targets. In addition, both benzobisthiol and coumarin derivatives inhibit the *S. aureus* and *B. subtilis* replicative helicases, by blocking the interaction of helicase with the double-stranded DNA substrate [108,112]. It is encouraging that some of the compounds identified in these studies also inhibited the growth of ciprofloxacin-resistant *S. aureus* [112], since this demonstrates that in vitro assays can identify compounds with the ability to enter some bacterial cells. Finally, 1,3,5-triaminotriazines were identified as inhibitors of the *P. aeruginosa* DnaB helicase in a large screen of 230,000 commercially-available small molecules [113]. Although these compounds showed some activity against *S. aureus*, they were unacceptably toxic to mammalian cells, and so were not considered as lead compounds for antibiotic development.

### 4.3. Cell Based Screen for Inhibitors of Bacterial Replication Initiation

Two cell-based assays [102,103] were developed to screen for the inhibitors of *V. cholerae* by targeting RctB, the essential initiator protein that triggers initiation from vc*oriC2*, the origin of chromosome 2 [16]. In one screen, vc*oriC2* was cloned and used as the sole replication origin of a drug-resistance plasmid in *E. coli* cells that have been engineered to express RctB. Vc*oriC2* is functional in *E. coli* as long as RctB is supplied, since *E. coli* replication proteins can successfully carry out helicase loading, priming and DNA replication [103,114]. 

Any compound that specifically inhibits RctB will prevent plasmid replication without affecting other proteins in the cell, causing the loss of the plasmid and its associated drug resistance determinant [102]. Parallel screening of isogenic plasmid-less cells identified compounds with other mechanisms of toxicity. Using this assay to screen 138,000 small molecules, a RctB inhibitor, termed vibrepin (3-(3,4-dichlorophenyl) cyclopropane-1,1,2,2-tetracarbonitrile), was identified [102]. However, when vc*oriC2* was inserted onto the chromosome of *E. coli* (expressing RctB) to drive chromosome replication in place of the native *E. coli oriC*, vibrepin did not inhibit the cell growth of the engineered cells [103]. This result is consistent with literature that shows that mutations in *E. coli* that inactivate cloned *oriC* are often tolerated by chromosomal *oriC* [81,115]. Thus, screens for inhibitors of replication initiation may be more successful if targeted toward chromosomal rather than cloned origins.

Cell-based assays for inhibitors of *E. coli oriC* have also been reported. One of these [101] is minichromosome based, and, like the plasmid-based RctB assay described above, it makes use of cells initiating from a chromosomal replication origin that is different than that used to trigger replication of the minichromosome. In this assay, the chromosomal copy of *oriC* is deleted, and the *E. coli* cells are forced to use constitutive Stable DNA Replication (cSDR) [116] in order to duplicate their chromosomes. The engineered cells also contain a reporter GFP gene inserted onto the chromosome, and an *oriC*-containing minichromosome carrying a gene encoding a transcriptional repressor of the GFP promoter. Inhibitors of initiation from *oriC* should cause an eventual loss of the minichromosome and the repressor gene, resulting in the activation of the GFP reporter. Although a screen of 400 natural product compounds using this assay did not yield any replication inhibitors [101], the assay has been validated using known repressors of DnaA, and it is possible that screening a larger library would result in some lead compounds. However, the same concerns related to plasmid and chromosomal *oriC* function also apply to this assay.

Two cell-based assays to identify agents that target initiation from chromosomal *E. coli oriC* have also been reported [100,104]. Both assays use cells that initiate more than once per cycle (over-initiation), and exploit the fact that over-initiation can be lethal, most likely because head-to-tail (co-directional) replication fork collisions can occur when closely spaced forks stall, causing catastrophic double-strand breaks [117]. The first assay utilizes cells carrying the *dnaA*(cos) allele [100]. DnaA(cos) carries two amino acid substitutions, one that prevents nucleotide binding (A184V), and another that most likely stabilizes the mutated form (Y271H) [118]. Cells harboring *dnaA*(cos) are non-viable at 30 °C because they over-initiate. The second assay [104] uses cells that over-initiate due an increased level of DnaA-ATP, which can be lethal in rich media, due to fork collision when replication is stalled by oxidative DNA damage [119]. Higher DnaA-ATP levels were caused by a mutation in the *hda* gene and a loss of RIDA (described above), or by excess copies of the genetic loci, DARS, that mediates the rejuvenation of DnaA-ATP from DnaA-ATP [36]. In both assays, any agent that reduces the chance of catastrophic fork collision, including those that decrease the initiation frequency and those that limit DNA damage and increase fork processivity, will suppress the lethal phenotypes and allow growth under non-permissive conditions (unless the compound is otherwise toxic to cells) [120]. The DnaA(cos)-based assay was used to screen 1400 small molecules from the Sigma Aldrich Library of Pharmacologically Active Compounds (LOPAC), and a benzazepine (6-chloro-7,8-dihydroxy-1phenyl-2,3,4,5-tetrahydro-1H-3-benzazepine) was identified as active [121]. Interestingly, this compound does not target DnaA or other proteins directly involved in initiation. Rather, it is reported to be a weak inhibitor of DNA gyrase [121], and so it could inhibit initiation by decreasing the negative superhelicity of the origin region, thus making it harder to unwind the DUE [122]. Alternatively, this compound is also reported to affect iron homeostasis, and thus it could prevent lethal over-initiation by limiting reactive oxygen species (ROS), which can cause oxidative DNA damage [104]. (ROS are produced when intracellular iron encounters hydrogen peroxide, which is produced by cells or in the environment) [123]. The DARS/had-based assay screened 400 microbial extracts, and found a compound, deferoxamine, which is a known iron chelator [104].

Although the assays described above provide an interesting starting ground, given the high level of diversity in bacterial origin configurations [95], screens for inhibitors of initiation from *E. coli oriC* might not be sufficient to identify agents that act against a broad range of bacteria. It seems likely that the development of successful screens will have to utilize multiple types of bacteria, or use heterologous systems. Pertinent to this, the concept of heterologous origin transplantation, where origins of one bacterial type are inserted into the *E. coli oriC* locus (replacing the native origin) was described in a recent review [77]. Heterologous origin transplantation (with or without the transplantation of heterologous replication proteins) could be used to develop cell-based screens for inhibitors of non-*E. coli* orisome assembly pathways, and would be particularly useful to screen for agents that inhibit initiation in bacteria that are either highly pathogenic or that are difficult to culture.

### 4.4. New Strategies for Inhibiting Initiation Based on Orisome Assembly and Regulation Pathways

Although there are no known natural products that directly inhibit the initiation of chromosome replication, all bacteria have molecular mechanisms that regulate orisome assembly to ensure that chromosome replication begins only at the correct time, and only once, per cell cycle [92]. Precise timing of chromosome replication is critical for bacterial genome stability, since under-initiation can lead to eventual chromosome loss, while over-initiation can result in replication fork collapse and genome instability [117]. Since mechanisms to avoid over-initiation depend on negative regulators to prevent an untimely completion of orisome assembly, they provide proof-of-principle that replication onset can be inhibited by targeting specific features of orisomes.

The most common method used by bacteria to control orisome assembly is to limit access of DnaA to *oriC*. Studies using *E. coli* reveal that much of this type of regulation is focused on DnaA binding to low affinity sites [124], both before and immediately after initiation [76]. Before initiation, the DNA bending protein Fis helps to maintain the origin in a conformation that prevents IHF from promoting cooperative binding between R1 and R5M (see above), until levels of DnaA increase enough to displace Fis from its recognition site [81,125,126]. Also, the presence of DnaA-ATP sites in *E. coli oriC* limits DnaA binding and orisome completion until DnaA-ATP levels rise to a critical level [83,126]. After initiation, 5′-GATC motifs in *oriC,* located in the DUE as well as in low affinity DnaA binding sites [80], are blocked by SeqA. While many bacteria appear to lack DnaA-ATP binding sites or orthologs to SeqA, they do have other factors to inhibit DnaA occupation. For example, *H. pylori* uses DNA topology to regulate low affinity DnaA/*oriC* interactions [55]. In *B. subtilis*, several proteins (including YabA, DnaD, and Soj) negatively regulate initiation by inhibiting the cooperative binding of DnaA at *oriC* [127,128,129,130,131]. Additional negative regulators of orisome formation include response regulators CtrA, MtrA and HP1021, which inhibit the DnaA occupation of *oriC* in *C. crescentus*, *M. tuberculosis* and *H. pylori*, respectively [54,132,133].

Based on the current knowledge of how DnaA binding to *oriC* is directed, and on how the factors described above exert their influence on orisome assembly, there are several possible modes of action by which small molecules could inhibit DnaA’s access to origin recognition sites. First, a compound could block domain IV-DNA (recognition site) interactions, either by interacting with key domain IV amino acids, or by associating directly with R-box-like sequences in DNA. Second, oligomerization of DnaA via either domain I or domain III interactions could be interrupted, preventing cooperative binding between high and low affinity sites in double-stranded origin DNA, and preventing binding to DnaA-ATP sites and single-stranded DNA at DnaA-trio elements and/or the DUE. It also seems probable that preventing DnaA-DnaB, DnaB-DnaB and DnaB-DnaC would be effective in preventing initiation.

One challenge to the strategies proposed above is that neither protein-DNA interactions nor protein-protein interactions have traditionally been considered to be easily “druggable”. However, this concept has been challenged recently, and small molecule inhibitors of the binding of transcription factors to DNA, interacting with both recognition site DNA and DNA binding domain targets, have been identified [134,135,136]. Also promising are recent studies demonstrating that interactions between subunits of the sliding clamp (DnaN) can be inhibited by cyclic peptides [137] and small molecules [138], and interactions between DnaN and the replicative DNA polymerase, Pol III, can be inhibited by small molecules [139] and by natural products [140].

Another challenge that pertains to all new antibiotics is how to delay the development of resistance. Past history indicates that sooner or later, bacterial cells will acquire mutations that confer resistance to any new antibiotic. However, even if resistance is unavoidable, it could be beneficial to target functions carried out by the most conserved regions of DnaA and DnaB, since this may also reduce the likelihood that mutations conferring resistance could arise without detrimentally affecting initiation and cell reproduction. There is also evidence that many of the most successful antibiotics delay resistance development by targeting more than one protein [141], but this may not be possible with effective inhibitors of initiation, particularly if the agents are targeted to specific functional regions of initiation proteins. There are, however, different strategies, such as hybrid drugs or combination chemotherapy, that would fulfill the same purpose of hitting more than one target during treatment.

The identification of inhibitors against targets previously considered to be undruggable is encouraging. We predict that, with continued development of in vitro and cell-based assays, and with large scale screening efforts, compounds that inhibit key aspects orisome assembly and function will be found. Further, the strategy of examining protein-protein interactions in unexploited pathways can be extended to other aspects of DNA biology, including the elongation phase of DNA replication [99,107] and DNA repair [142,143], and used to discover lead compounds to develop novel antibiotics to fight the scourge of drug-resistant bacterial infections.
